# Anticancer and Antioxidant Effects of Bioactive Peptides from Black Soldier Fly Larvae (*Hermetia illucens*)

**DOI:** 10.3390/nu17040645

**Published:** 2025-02-11

**Authors:** Kwanchanok Praseatsook, Arpamas Vachiraarunwong, Sirinya Taya, Phatthawin Setthaya, Kenji Sato, Hideki Wanibuchi, Rawiwan Wongpoomchai, Pornngarm Dejkriengkraikul, Min Gi, Supachai Yodkeree

**Affiliations:** 1 Department of Biochemistry, Faculty of Medicine, Chiang Mai University, Chiang Mai 50200, Thailand; kwanchanok_pa@cmu.ac.th (K.P.); rawiwan.wong@cmu.ac.th (R.W.); pornngarm.d@cmu.ac.th (P.D.); 2 Department of Environmental Risk Assessment, Osaka Metropolitan University Graduate School of Medicine, Osaka 545-8585, Japan; arpamas.vachi@omu.ac.jp (A.V.); wani@omu.ac.jp (H.W.); 3 Functional Food Research Unit, Multidisciplinary Research Institute, Chiang Mai University, Chiang Mai 50200, Thailand; sirinya.t@cmu.ac.th; 4 Science and Technology Research Institute, Chiang Mai University, Chiang Mai 50200, Thailand; phathawin.l@cmu.ac.th; 5 Division of Applied Biosciences, Graduate School of Agriculture, Kyoto University, Kyoto 606-8502, Japan; sato.kenji.7x@kyoto-u.ac.jp; 6 Anticarcinogenesis and Apoptosis Research Cluster, Faculty of Medicine, Chiang Mai University, Chiang Mai 50200, Thailand

**Keywords:** black soldier fly larvae, insect protein hydrolysates, antioxidant activity, antimutagenicity, anti-inflammatory activity, anticancer activity, colon cancer

## Abstract

Background: Protein hydrolysates from insects are recognized for their biological activities. Black soldier fly larvae (BSFL) have drawn attention due to their antioxidant protein hydrolysates. However, research on bioactive peptides derived from these hydrolysates, particularly their cancer chemopreventive potential, remains limited. This study aims to evaluate the antioxidant, anti-inflammatory, antimutagenic, and anticancer activities of BSFL-derived bioactive peptides and explore the molecular mechanisms. Methods: Alkali-soluble BSFL protein (ASBP) was extracted and hydrolyzed using Alcalase and bromelain under optimized conditions. Antioxidant activity was assessed via FRAP, ABTS, and DPPH assays. The hydrolysate with the highest antioxidant activity was fractionated into molecular weight (MW) groups (>30, 10, and <3 kDa). The bioactivity of fractionated peptides was evaluated through antioxidant, anti-inflammatory (nitric oxide production in RAW 264.7 cells), antimutagenic (Ames test), and anticancer (CCK-8 assay on HCT 116, COLO205, Cw-2, and Caco-2 cells) assays. Mechanistic insights were obtained via microarray and Western blot analyses. Peptides were identified by LC-MS/MS. Results: The ASBP-Alcalase hydrolysate (ASBP-AH) showed optimal antioxidant activity at 3% (*w*/*w*) for 4 h. The ASBP-AH 30 (MW > 30 kDa) fraction exhibited the highest antioxidant capacity. In contrast, the ASBP-AH3 (MW < 3 kDa) fraction exhibited significant antimutagenic effects, reduced nitric oxide production, and decreased COLO205 cell viability. Treatment with ASBP-AH3 at its LC_50_ dose modulated the SKP2/p21/cyclin D1 pathways. Mostly peptides from ASBP-AH3 were composed of hydrophobic and charged amino acids. Conclusions: BSFL-derived bioactive peptides exhibit potential as multifunctional agents for cancer chemoprevention. *In vivo* studies are required to explore their clinical applications.

## 1. Introduction

Bioactive peptides offer significant advantages as therapeutic agents due to their high biological activity and cost-effectiveness in treatment. These peptides are typically associated with low toxicity, resulting in minimal side effects and the reduced risk of complications for patients [1]. These characteristics are influenced by the nature of the protein source, the specific enzyme used, and the hydrolysis conditions, including temperature and pH [2]. Various enzymes have been used to produce hydrolysates from protein sources. Enzymatic hydrolysis can be carried out using a variety of commercially available enzymes derived from plants, animals, and microorganisms. These enzymes catalyze the cleavage of peptide bonds at specific sites, promoting the formation of bioactive peptides [3]. Alcalase and bromelain are two of the most widely studied enzymes. Alcalase, an endopeptidase, is produced by *Bacillus licheniformis*, whereas bromelain, also an endopeptidase, is extracted from pineapple stems. Studies have demonstrated that the hydrolysis of proteins from sources such as pigeon pea, lentil, and chickpea using Alcalase and bromelain can significantly improve their antioxidant, and anti-inflammatory activities [4].

Bioactive peptides from the enzymatic hydrolysis of food protein sources have demonstrated anticancer activity. These peptides exhibit high selectivity to cancer cells while showing minimal toxicity to normal cells [5]. Their low molecular weight (MW) enhances intracellular transport and membrane interactions, leading to cancer cell death [6]. Additionally, bioactive peptides offer significant advantages as therapeutic agents due to their high biological activity and cost-effectiveness, making them promising candidates for anticancer therapy [7]. For example, a bioactive peptide derived from chickpeas has been shown to inhibit breast cancer cell proliferation by upregulating p53 expression [8]. Similarly, peptide fractions isolated from maize albumin hydrolysate induced apoptosis in liver cancer cells by downregulating the expression of anti-apoptotic factors [9]. Comparable results have been observed in studies involving common bean peptides and riceberry rice bran peptides [10,11]. Moreover, an overproduction of reactive oxygen species (ROS) can cause oxidative damage to biomolecules such as lipids, proteins, and DNA. This oxidative damage has been linked to the development of various diseases, including diabetes, cardiovascular diseases, and cancer [12]. Thus, antioxidant nutrition is believed to reduce the risk of free radical-related health diseases. Compared to the potential harm of synthetic antioxidants, natural antioxidants from natural sources have attracted extensive attention due to their wide range of sources and better safety [13]. Protein hydrolysates from various natural sources have shown an excellent capacity for scavenging free radicals like ROS, inhibiting lipid peroxidation and protein oxidation [14]. Edible insects are emerging as sustainable and viable alternative sources of nutrition [15]. Recent studies have highlighted the diverse biological activities of protein hydrolysates derived from various insect species. For example, silkworms (*Bombyx mori*) exhibit significant antioxidant and anti-inflammatory properties [16,17], while protein hydrolysates from grasshoppers (*Locusta migratoria*) demonstrate both antioxidant and antimicrobial activities [18,19]. Additionally, protein hydrolysates from silkworm pupae, produced using Alcalase, have shown anticancer potential by inhibiting cell proliferation and inducing apoptosis in cancer cell lines [20]. These findings emphasize the promising role of insect-derived protein hydrolysates as health-promoting alternatives to conventional protein sources.

Among the insects mentioned, black soldier fly larvae (*Hermetia illucens*, BSFL) have gained attention for their exceptional nutritional profile and sustainable cultivation [21]. The larval stage is particularly rich in proteins and bioactive compounds, making it an excellent source for peptide extraction [22]. Recent studies have demonstrated the strong antioxidant activity of BSFL protein hydrolysates generated through enzymatic hydrolysis using proteases such as Alcalase and bromelain [23,24]. Moreover, BSFL extract has been reported to exhibit antimicrobial activity against both Gram-negative and Gram-positive bacteria [25]. Many studies have investigated the antioxidant activity of BSFL protein hydrolysates. However, research on bioactive peptides derived from these hydrolysates remains limited, particularly regarding their antioxidant and anticancer properties. This present study aims to evaluate the antioxidant, anti-inflammatory, antimutagenic, and anticancer activities of these bioactive peptides using *in vitro* experiments. Additionally, the molecular mechanisms underlying their anticancer activity were explored.

## 2. Materials and Methods

### 2.1. Chemicals

Alcalase (3.018 U/mL) was obtained from Merck (Darmstadt, Germany). Sodium hydroxide (NaOH) and sodium chloride (NaCl) were purchased from RCI Labscan™ (Bangkok, Thailand). O-phthaldialdehyde (OPA) and L-serine, used to determine the degree of hydrolysis, were sourced from Merck (Burlington, MA, USA). Dithiothreitol (DTT) was obtained from Vivantis (Selangor Darul Ehsan, Malaysia). Bromelain (3 U/mL), aflatoxin B1 (AFB_1_), sodium azide (NaN_3_), lipopolysaccharides (LPS), 2,2′-azino-bis(3-ethylbenzothiazoline-6-sulfonic acid) (ABTS), 1,1-diphenyl-2-picrylhydrazyl (DPPH), ferric-reducing antioxidant power (FRAP), 2,4,6-tris(2-pyridyl)-s-triazine (TPTZ), and 6-hydroxy-2,5,7,8-tetramethylchroman-2-carboxylic acid (Trolox) were purchased from Sigma-Aldrich Corp. (St. Louis, MO, USA). Phenyl isothiocyanate (PITC), 2-amino-3,4-dimethylimidazo [4,5-f]quinoline (MeIQ), 2-aminoanthracene (2-AA), and 2-(2-furyl)-3-(5-nitro-2-furyl)-acrylamide (AF-2) were obtained from Wako Pure Chemicals (Osaka, Japan). Triethylamine (TEA) was purchased from Thermo Fisher Scientific Inc. (Waltham, MA, USA). Eagle’s Minimum Essential Medium (EMEM), Dulbecco’s Modified Eagle Medium (DMEM), and RPMI-1640 medium were supplied by FUJIFILM Wako Pure Chemical Corporation (Osaka, Japan). All other chemicals used were of analytical grade.

### 2.2. Preparation of Alkali-Soluble BSFL Protein

BSFL powder, obtained after the separation of the oil fraction, was kindly provided by EXOFOOD THAILAND CO., Ltd. (Bangkok, Thailand). Hexane was used to completely defat the BSFL powder at a solvent-to-sample ratio of 5 mL/g. The mixtures were centrifuged at 6000 rpm for 15 min, and the defatted pellets were retained for proximate analysis. The solvents from the supernatant fractions were evaporated. Defatted BSFL powder was soaked in 0.5 M of NaOH and stirred for 2 h using a mixer (IKA^®^ RW 20 digital, Staufen, Germany). The solutions were then centrifuged at 6000 rpm for 15 min, and the supernatants were collected. The pH of the supernatants was adjusted to 4.8 using 6 M of HCl to precipitate the alkali-soluble BSFL protein (ASBP). The resulting ASBP was stored at −20 °C for the further preparation of ASBP hydrolysates (ASBP-H).

### 2.3. Preparation of ASBP-H

#### 2.3.1. Optimization of ASBP-H Conditions Using Antioxidant Assays

The effects of enzyme concentrations and incubation times on ASBP hydrolysates (ASBP-H) were examined to investigate the antioxidant activities of individual Alcalase and bromelain enzymes, which were selected based on their ability to generate bioactive peptides with antioxidant properties in previous studies [23,24]. To obtain ASBP-Alcalase hydrolysates (ASBP-AHs) and ASBP-bromelain hydrolysates (ASBP-BHs), ASBP was hydrolyzed with each enzyme at enzyme/substrate ratios (%E/S) ranging from 0 to 4:100 (*w*/*w*) at pH 8. Incubation times ranged from 1 to 8 h and 6–48 h at temperatures of 55 °C and 50 °C for Alcalase and bromelain, respectively. The criteria for selecting the optimal hydrolysis conditions were also based on the guidance from the aforementioned studies. After hydrolysis, the reactions were terminated by heating at 90 °C for 10 min. The hydrolysates were centrifuged at 6000 rpm for 15 min, and the supernatants were collected, lyophilized, and stored at −20 °C for subsequent antioxidant assays. To evaluate the combined effects of Alcalase and bromelain enzymes on antioxidant activities, the optimal hydrolysis conditions for each enzyme (identified from initial tests) were applied sequentially. After hydrolysis with the first enzyme, the reaction was stopped by heating at 90 °C for 10 min, and the temperature was then adjusted to the conditions suitable for the second enzyme. When ASBP was hydrolyzed with Alcalase followed by bromelain, the resulting product was designated as ASBP-AH+ASBP-BH. Conversely, when bromelain was used first, followed by Alcalase, the product was referred to as ASBP-BH+ASBP-AH.

#### 2.3.2. Isolation of ASBP-AH

ASBP-AH was dissolved in distilled water and added to centrifuge tubes with MW cutoffs of 3, 10, and 30 kDa (Amicon^®^ Ultra-15 Centrifugal Filter Unit, Merck, Darmstadt, Germany). The solution was then centrifuged at 5000 rpm and 4 °C for 30 min to obtain ASBP-AH fractions, including ASBP-AH30 (MW > 30 kDa), ASBP-AH10–30 (MW 10–30 kDa), ASBP-AH3–10 (MW 3–10 kDa), and ASBP-AH3 (MW < 3 kDa). These fractions were lyophilized and stored at −20 °C for further analysis.

### 2.4. Characterization of ASBP and ASBP-H

#### 2.4.1. Nutritional Composition Using Proximate Analysis

The protein content of BSFL residues was determined using a nitrogen/protein analyzer (Leco Inc. FP-528, St. Joseph, MI, USA). In accordance with AOAC guidelines [26], the nitrogen combustion method was used, with a nitrogen-to-protein conversion factor of 5.62 for BSFL [27]. Moisture content was measured using a moisture analyzer (Presisa XM60, Zurich, Switzerland). Fat content was determined using a Soxhlet extraction system (SOXTECTM 8000, FOSS, Bangkok, Thailand). Ash content was measured by heating the sample in a furnace at 600 °C for 6 h in an ash oven (MKF-5, Mikrotest, Istanbul, Turkey). The values were expressed as a percentage of dry weight. Proximate analysis was conducted at the Science and Technology Research Institute, Chiang Mai University.

#### 2.4.2. Amino Acid Analysis

The total amino acid composition was determined by reversed-phase high-performance liquid chromatography (RP-HPLC) using an L-column3 C18 (4.0 × 250 mm, Shimadzu, Kyoto, Japan) according to the Edman method [28]. Glass tubes were filled with 10 μL of a 10 mg/mL sample stock. The samples were then dried under vacuum, digested with 6 M of HCl, and incubated at 150 °C for 1 h. After neutralizing the acidic solution, the alkaline environment was modified for PITC activity. A 20 μL PITC solution, composed of methanol, water, and TEA in a 7:1:2 ratio, was added, and the mixture was incubated at room temperature for 30 min before being redried under vacuum. Each sample was then mixed with 200 μL of PICO Tag buffer. The samples were sonicated for 20 s and filtered using a syringe filter. After 5 min of centrifugation at 120 rpm and 4 °C, 100 μL of the supernatant was collected and analyzed by RP-HPLC.

#### 2.4.3. Determination of ASBP and ASBP-H Pattern

ASBP and ASBP-H were fractionated using 15% running and 4% stacking gels in one-dimensional sodium dodecyl sulfate polyacrylamide gel electrophoresis (SDS-PAGE) [29]. Electrophoresis was performed in an SDS-running buffer at a constant current of 250 V (30–40 A). Tris-glycine was used as the electrode buffer for peptides with a high MW, while tris-tricine was used for peptides with a MW between 2 and 40 kDa. After electrophoresis, the gels were stained for 15 min with 0.25% Coomassie Brilliant Blue R-250, followed by destaining in methanol and acetic acid for 8 h. The destaining process continued until the background was transparent enough for band scanning.

#### 2.4.4. Determination of Degree of Hydrolysis (DH)

Following Setthaya et al., the method and formula for calculating the DH of ASBP-H used in this study were described [30]. ASBP-H was digested with 6 N of HCl for 18 h at 110 °C, adjusted to pH 7 with 6 N of NaOH, and then diluted with distilled water. To determine the peptide content produced after the hydrolysis process, an OPA reagent was added to the hydrolysate solution. The mixture was measured at 340 nm. Using the L-serine standard curve, the peptide content of the hydrolysate samples was calculated. The value is expressed as mg of L-serine per mL of sample.

### 2.5. Biological Activities of ASBP-AH and Its Fractions

#### 2.5.1. Determination of Antioxidant Activity via Colorimetric Techniques

DPPH and ABTS assays were performed to evaluate the radical scavenging capacity. Hydrolysate samples (10 mg/mL) were incubated with a 0.2 mM DPPH solution for 0 and 30 min at room temperature in the dark, and absorbance was measured at 517 nm [30]. The ABTS^•+^ radical was generated by reacting 7 mM of ABTS with 2.45 mM of potassium persulfate (1:1) and left in the dark for 12–16 h before use. Samples were incubated with the diluted ABTS^•+^ solution for 30 min, and absorbance was recorded at 734 nm. The FRAP assay was used to assess ferric reducing capacity using the same sample concentrations. Fresh reagent was prepared by mixing a 300 mM sodium acetate buffer, 10 mM of TPTZ, and 20 mM of ferric chloride (10:1:20) and reacted with samples at 37 °C for 30 min in the dark. Absorbance was measured at 593 nm [30]. Antioxidant activities were expressed as Trolox equivalent antioxidant capacity (TEAC) based on Trolox standard curves.

#### 2.5.2. Determination of Antimutagenicity Using the Salmonella Mutation Assay

The Ames test, a well-established screening method for assessing the mutagenic potential of bioactive compounds [31], was conducted on ASBP-AH and its fractions using *Salmonella typhimurium* strains TA98 (frame–shift mutation) and TA100 (base–pair substitution), which were provided by Dr. Kei-Ichi Sugiyama (National Institute of Health, Tokyo, Japan). Samples (1 and 5 mg/plate) were tested with and without metabolic activation (±S9 mix) at pH 7.4. After pre-incubation at 37 °C for 20 min, top agar with 0.5 mM of L-histidine/D-biotin was added, and the mixture was plated on minimal agar, followed by 48 h of incubation. AFB_1_ and AF-2 were used as positive controls for TA98 under +S9 and −S9 conditions, respectively, while MeIQ and NaN_3_ served as positive controls for TA100. A substance was considered mutagenic if revertant colonies doubled relative to the vehicle control [32]. For antimutagenicity, 1 mg/plate of each sample was tested with ±S9, using dimethyl sulfoxide or distilled water as negative controls. Positive controls included AFB_1_ (+S9) and AF-2 (−S9) for TA98, and MeIQ (+S9) and NaN_3_ (−S9) for TA100. After pre-incubation (20 min), samples were plated with top agar on minimal glucose agar and incubated for 48 h at 37 °C. Revertant colonies were counted, and inhibition percentages were calculated as described by Guo et al. [32].

#### 2.5.3. Determination of Anti-Inflammatory Activities in Murine Macrophages

To determine the anti-inflammatory activity of ASBP-AH and its fractions, 2 × 10^4^ cells per well of RAW 264.7 macrophages (TIB-71™; American Type Culture Collection, ATCC) were cultured in DMEM supplemented with 10% fetal bovine serum (FBS) and 1% penicillin/streptomycin. The cells were incubated for 24 h at 37 °C with 5% CO_2_ before being treated with various concentrations of ASBP-AH and its fractions for 2 h. Subsequently, the macrophage cells were stimulated for 24 h with 1 μg/mL of LPS. Then, 100 μL of cell supernatants were transferred to 96-well plates. The next step involved adding the Griess reagent, which consisted of 0.1% N-1-naphthylenediamine dihydrochloride, 1% sulfanilamide, and 2.5% phosphoric acid. The optical density was measured at 550 nm, and the nitrite concentration was determined using a standard curve of sodium nitrite [32].

#### 2.5.4. Evaluation of Cytotoxicity

Cell viability was assessed to determine the cytotoxicity of ASBP-AH and its fractions against normal fibroblast and colon cancer cell lines. Fibroblasts (PCS-201-013, ATCC) and HCT 116 cells (RCB2979, RIKEN BRC Cell Bank) were cultured in DMEM with 10% FBS, while COLO205 (RCB2127) and Cw-2 (RCB0778) cells were maintained in RPMI-1640 with 10% FBS. Caco-2 cells (RCB0988) were cultured in EMEM with 20% FBS. All media contained 1% penicillin/streptomycin. Cells were seeded into 96-well plates (5 × 10^3^ cells/well) and incubated for 24 h. The medium was then replaced with ASBP-AH and its fractions (100–400 μg/mL) and incubated for an additional 24 h. Cell viability was measured using the Cell Counting Kit-8 (CCK-8) assay [33], and LC_50_ values were calculated by nonlinear regression analysis using GraphPad Prism 10.3.1 [34].

### 2.6. Microarray Analysis

Total RNA was extracted from a 6-well plate (5 × 10^5^ cells/well) using the RNeasy Mini Kit (Qiagen, Hilden, Germany) following a 24 h treatment of COLO205 with an LC_50_ dose of ASBP-AH3. The microarray analysis was performed by Cell Innovator Inc. (Fukuoka, Japan) using the Affymetrix Clariom D Assay, Human (Affymetrix, Inc., Santa Clara, CA, USA). Raw signal intensities were normalized using the SST-RMA and quantile algorithms with Affymetrix Expression Console 1.1 software. To identify upregulated and downregulated genes, z-scores and ratios were calculated from the normalized signal intensities of each probe compared to the control. The criteria for identifying altered genes were described by Vachiraarunwong et al. [33]. The functional annotation and pathway analysis of differentially expressed genes were performed using Ingenuity Pathway Analysis (IPA, Ingenuity Systems, Inc., Redwood City, CA, USA). Pathway summaries were generated using z-scores and *p*-values obtained from the Bioinformatics platform [35] and visualized as a heatmap with GraphPad Prism version 10 (GraphPad Software, San Diego, CA, USA). Additionally, protein–protein interaction networks were constructed using STRING version 12.0 [36].

### 2.7. Western Blot Analysis

Total protein was extracted from COLO205 cells (5 × 10^5^ cells/well in 6-well plates) treated with the LC_50_ dose of ASBP-AH3 for 24 h, following the protocol by Vachiraarunwong et al. [33]. Protein samples (20 μg) were mixed with 6X sample buffer, heated to 95 °C for 5 min, and separated by 12.5% SDS-PAGE, followed by transfer to Amersham™ Hybond™ PVDF membranes (GE Healthcare, Chicago, IL, USA). The membrane was blocked with 5% skim milk in 0.1% Tris-buffered saline with Tween20 for 1 h and incubated with primary antibodies overnight at 4 °C. Primary antibodies included anti-Skp2 (1:1000), anti-p21 Waf1/Cip1 (1:1000), anti-Cyclin D1 (1:1000) (Cell Signaling Technology, Danvers, MA, USA), and anti-β-actin (1:50,000, Santa Cruz Biotechnology, Dallas, TX, USA). After washing, the membrane was incubated with goat anti-rabbit IgG-HRP secondary antibody (1:10,000, Cell Signaling Technology, Danvers, MA, USA) for 1 h. Protein bands were visualized using SuperSignal™ West Pico PLUS (Thermo Scientific, Waltham, MA, USA) and imaged with Fusion SOLO.7S (Vilber Lourmat, Collégien, France).

### 2.8. The Identification of Peptide Sequences Using LC-MS/MS

The freeze-dried ASBP-AH3 was sent to Ward Medic Ltd., Part (Bangkok, Thailand) for peptide sequencing via LC-MS/MS. The sample was dissolved in 0.1% formic acid in LC water (89 ng/μL), and 10 μL was injected into a 25 cm Easy-Spray C18 column. Peptide analyses were performed using an EASY-nano LC 1000 system and a Q-Exactive™ Plus Hybrid Quadrupole-Orbitrap™ mass spectrometer at 2.0 kV. The mobile phase consisted of 0.1% formic acid in water (A) and acetonitrile (B), with the following gradient: 0 min, 5% B; 50 min, 30% B; 80 min, 50% B; 82 min, 98% B; and 90 min, 98% B. Full MS scans were acquired at a resolution of 70,000 (350–1400 *m*/*z*) with an AGC target of 3 × 10^6^ ions and a maximum IT of 250 ms. MS/MS spectra were obtained at a resolution of 17,500 with an AGC target of 5 × 10^4^ ions and a maximum IT of 100 ms. A 1.2 *m*/*z* isolation window and 10 loop counts were used. Fragmentation was performed by HCD with a normalized collision energy (NCE) of 27.

### 2.9. De Novo Peptide Sequencing

Data analysis was performed using the Global Proteome Machine database (www.thegpm.org/crap, accessed on 28 August 2024). The search parameters included trypsin digestion with up to three missed cleavages, the variable oxidation of methionine, and the fixed carbamidomethylation of cysteine (+57.02146 Da). A mass tolerance of 10 ppm for precursor ions and 0.02 Da for fragment ions was applied. The false discovery rate (FDR) was set at 1% using Q-values to ensure high-confidence peptide identification. Data processing and analysis were carried out using PEAKS Studio software version 10.6 (Bioinformatics Solutions Inc., Waterloo, ON, Canada).

### 2.10. Statistical Analysis

All results are presented as mean ± standard deviation (n = 3) using SPSS^®^ software version 27.0 (SPSS Inc., Chicago, IL, USA). One-way analysis of variance (ANOVA) was used to detect statistically significant differences among various samples (*p* < 0.05). Duncan’s multiple range test (DMRT) was applied to determine significant differences among the groups. For Western blot data, a Student’s *t*-test was used to assess differences between two groups using GraphPad Prism version 10.3.1 (GraphPad Software, San Diego, CA, USA), with significance set at * *p* < 0.05 and ** *p* < 0.01.

## 3. Results

### 3.1. ASBP Extracted from BSFL

**Figure 1:**
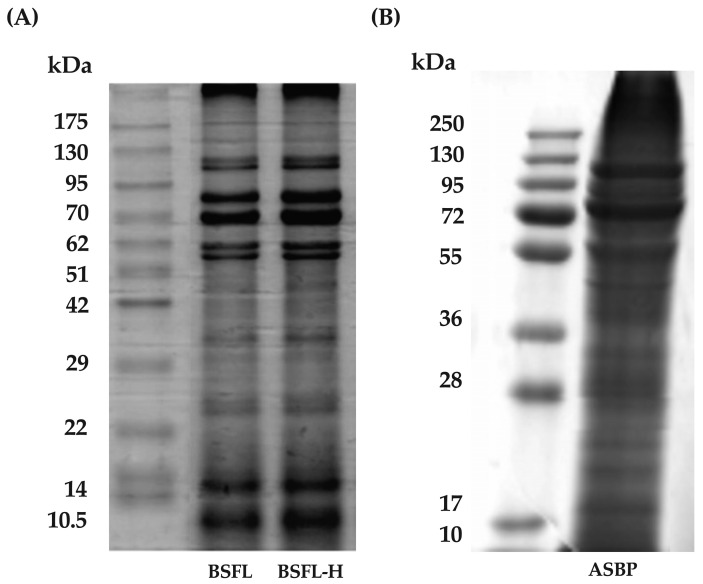
(**A**) MW distribution of BSFL and BSFL-H; and (**B**) MW distribution of ASBP.

Protein extraction from insects using alkaline solutions has been reported to yield the highest protein content [37]. In this study, BSFL powder was defatted using hexane to obtain a pellet known as the BSFL-H fraction. This fraction was then soaked in 0.5 M of NaOH to solubilize the proteins. The soluble proteins were subsequently precipitated with HCl, resulting in the ASBP fraction. The chemical composition analysis indicated that BSFL contained a higher fat content compared to BSFL-H and ASBP, whereas ASBP exhibited the highest protein and ash content (Table 1). The MW distribution of the extracts was analyzed using gel electrophoresis. Figure 1A,B illustrate the MW profiles of BSFL, BSFL-H, and ASBP. No significant differences in MW distribution were observed between BSFL and BSFL-H, with nine prominent bands detected at approximately 10.5, 14, 22–29, 29–42, 51–62, 62, 70, 70–95, and 95–130 kDa. In contrast, ASBP displayed a distinct MW profile, featuring four main bands at approximately 36–55, 55, 72, and 95–130 kDa. These results suggest that the alkaline extraction method improved protein yield and reduced lipid content while preserving key MW bands associated with functional protein fractions.

### 3.2. Optimization of the Enzymatic Hydrolysis for ASBP Peptide Production

**Figure 2:**
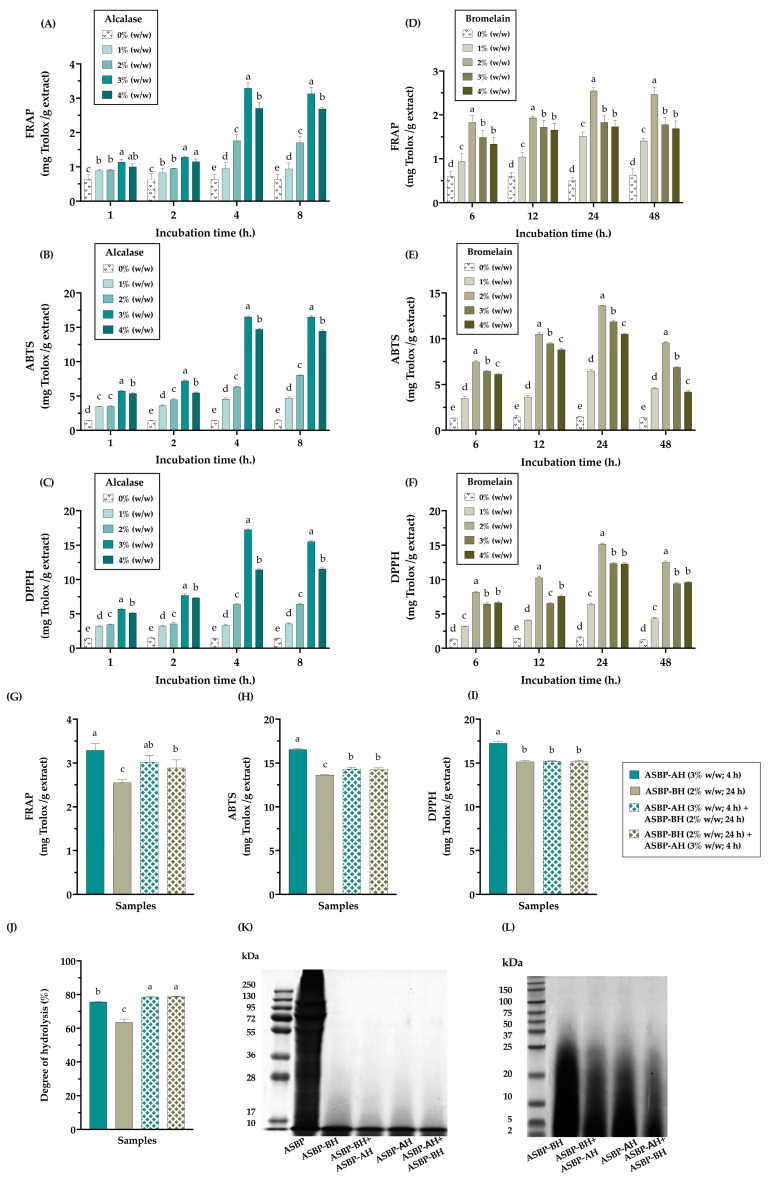
Antioxidant activities and peptide characteristics of ASBP-H under different enzyme concentrations and incubation times: (**A**–**C**) antioxidant activities of ASBP-Alcalase hydrolysates (ASBP-AH) under varying enzyme concentrations (% *w*/*w*) and incubation times (h); (**D**–**F**) antioxidant activities of ASBP-bromelain hydrolysates (ASBP-BH) under varying enzyme concentrations (% *w*/*w*) and incubation times (h); (**G**–**I**) effect of ASBP-AH and ASBP-BH combinations on antioxidant hydrolysate production; (**J**) %DH of individual ASBP-AH and ASBP-BH treatments and their combination; (**K**) SDS-PAGE analysis using Tris-glycine as the electrode buffer to resolve peptides with high MWs; (**L**) SDS-PAGE analysis using Tris-tricine as the electrode buffer to resolve peptides with low MWs. Antioxidant activities are expressed as mg Trolox equivalent per gram of extract. Values are presented as mean ± SD (n = 3). Statistical significance determined using one-way ANOVA followed by Duncan’s multiple range test; distinct letters indicate statistically significant differences between groups (*p* < 0.05).

Alcalase and bromelain have been widely used to produce bioactive peptide hydrolysates from various protein sources. In this study, the conditions for enzymatic hydrolysis, including process duration and enzyme concentration, were optimized. The antioxidant properties of the hydrolysates were evaluated using FRAP, ABTS, and DPPH assays. Figure 2A–F demonstrate that ASBP-AH exhibited the highest antioxidant activities at a concentration of 3% (*w*/*w*) after 4 h of incubation. In contrast, ASBP-BH showed the highest antioxidant activities at a concentration of 2% (*w*/*w*) after 24 h of incubation. Based on these findings, ASBP-AH and ASBP-BH at their optimal concentrations and durations were selected to evaluate the effect of enzyme combinations on antioxidant activities. As shown in Figure 2G–I, single ASBP-AH demonstrated higher antioxidant activity, particularly in the ABTS and DPPH assays, compared to single ASBP-BH and the enzyme combination groups ASBP-AH+ASBP-BH and ASBP-BH+ASBP-AH. Moreover, the DH was assessed as the percentage of cleaved peptide bonds after enzymatic hydrolysis [38]. The results showed that single-enzyme treatments exhibited a lower DH compared to enzyme combination groups, with DH values recorded as ASBP-AH (75.64 ± 0.26%) and ASBP-BH (63.56 ± 1.81%), while ASBP-AH+ASBP-BH (78.55 ± 0.41%) and ASBP-BH+ASBP-AH (78.74 ± 0.15%) showed higher DH values (Figure 2J). These findings indicate that the combination of Alcalase and bromelain was more efficient in cleaving peptide bonds. However, SDS-PAGE analysis using a Tris-glycine buffer to investigate the MW distribution of the protein hydrolysates revealed that all hydrolysates exhibited smear bands in the 10–20 kDa range, in contrast to ASBP (Figure 2K). Additionally, SDS-PAGE analysis under Tris-tricine conditions showed that the MW distribution profile of ASBP-BH exhibited more intense protein bands within the MW range of 10–37 kDa compared to ASBP-AH and the enzyme combination groups. However, ASBP-AH and the enzyme combination groups displayed smear bands in the MW range above 10 kDa and intense bands below 10 kDa (Figure 2L). These results suggest that hydrolyzing ASBP with Alcalase at 3% (*w*/*w*) for 4 h is the optimal condition for producing the most effective antioxidant peptides. This method successfully breaks down native proteins into short peptide fragments and enhancing antioxidant activity.

### 3.3. Antioxidant and Anti-Inflammation of ASBP-AH Peptide Fractions

**Figure 3:**
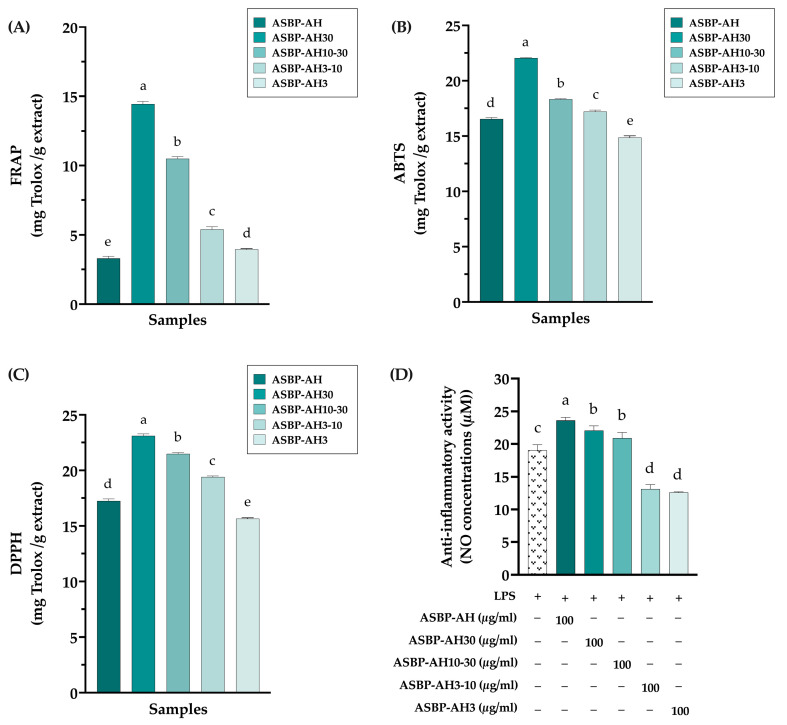
Effects of ASBP-AH peptide fractions on antioxidant and anti-inflammation: The antioxidant activity of ASBP-AH fractions tested by FRAP (**A**), ABTS (**B**), and DPPH (**C**) assay. Antioxidant activities are expressed as mg Trolox equivalent per gram of extract. (**D**) Anti-inflammatory activities of ASBP-AH and its fractions in 1 μg/mL LPS-induced RAW 264.7 cell lines. Values are presented as mean ± SD (n = 3). Statistical significance determined using one-way ANOVA followed by Duncan’s multiple range test; distinct letters indicate statistically significant differences between groups (*p* < 0.05).

According to previous studies, peptide hydrolysates with MWs less than 3 kDa exhibit the highest antioxidant activities [23,39]. Based on this finding, the peptides from ASBP-AH were fractionated using ultrafiltration into four MW ranges: greater than 30 kDa (ASBP-AH30), between 10 and 30 kDa (ASBP-AH10-30), between 3 and 10 kDa (ASBP-AH3-10), and less than 3 kDa (ASBP-AH3). The antioxidant and anti-inflammatory activities of these fractions were subsequently evaluated. As shown in Figure 3A–C, ASBP-AH30 exhibited the highest antioxidant activity across FRAP, ABTS, and DPPH assays. In contrast, ASBP-AH3 displayed the lowest radical scavenging capacity, although its activity was comparable to that of unfractionated ASBP-AH. Conversely, ASBP-AH fractions with MWs less than 10 kDa significantly reduced LPS-induced nitric oxide (NO) production in RAW macrophage cells at a concentration of 100 μg/mL, compared to higher MW fractions (Figure 3D). These results suggest that the peptides in ASBP-AH exhibit promising bioactive properties, with both antioxidant and anti-inflammatory activities influenced by peptide size.

### 3.4. Antimutagenicity Activity of ASBP-AH Peptide Fractions

**Figure 4:**
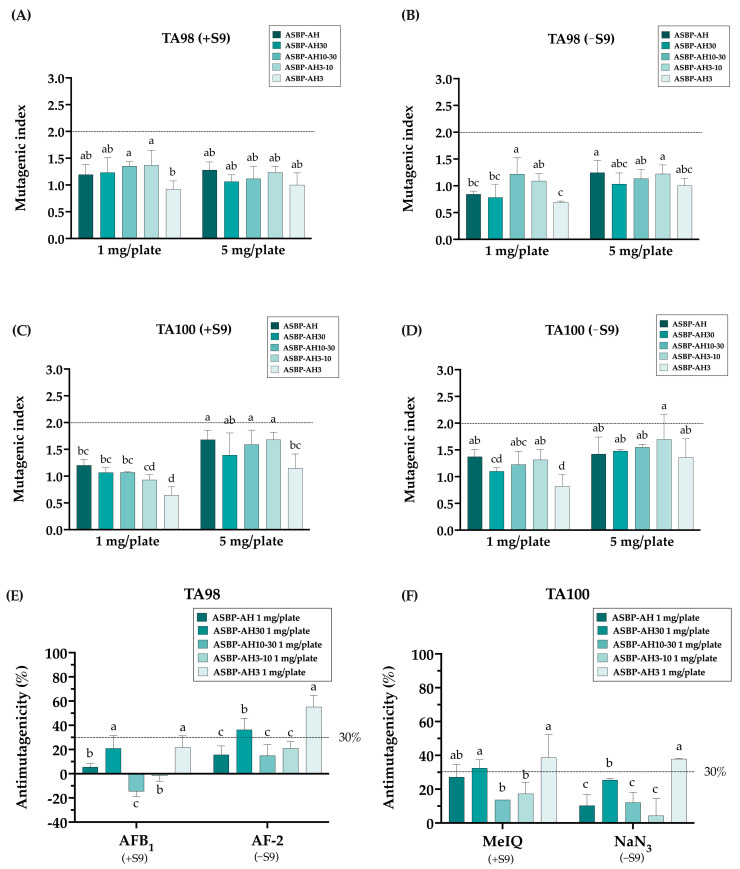
Mutagenic and antimutagenic activity of ASBP-AH peptide fractions: (**A**,**B**) Mutagenic activity of ASBP-AH peptide fractions in TA98 strain with (**A**) or without S9 (**B**). (**C**,**D**) Mutagenic activity in TA100 strain with (**C**) or without S9 (**D**). (**E**,**F**) Antimutagenic activity of ASBP-AH peptide fractions (1 mg/plate) against mutagens: AFB_1_ with S9 and AF-2 without S9 in TA98 strain (**E**), and MeIQ with S9 and NaN_3_ without S9 in TA100 strain (**F**). The values are expressed as mean ± SD (n = 3). According to a one-way ANOVA with Duncan’s multiple range test, distinct letters indicate statistically significant differences between groups (*p* < 0.05).

The ASBP-AH fractions were evaluated for their potential antimutagenic activity using the Ames test. To determine the mutagenicity of ASBP-AH fractions, both *Salmonella typhimurium* strains TA98 and TA100 strains were incubated with ASBP-AH and its fractions, with or without metabolic activation (+S9). As shown in Figure 4A–D, ASBP-AH and its fractions at concentrations of 1 and 5 mg/plate did not increase the reversal of mutations in either strain, as indicated by a mutagenic index (MI) of less than 2. The antimutagenic activity of ASBP-AH fractions was subsequently investigated against mutagens, including AFB_1_, AF-2, MeIQ, and NaN_3_. As shown in Figure 4E,F, the incubation of TA98 and TA100 strains with ASBP-AH3 at 1 mg/plate demonstrated the strongest antimutagenic activity, reducing AF-2 mutagenicity by 55.19% in TA98 and NaN_3_ mutagenicity by 37.87% in TA100. In contrast, ASBP-AH30 exhibited moderate inhibition, reducing AF-2-induced mutagenesis by 36.34% in TA98. However, treatment with ASBP-AH fractions under +S9 conditions in TA98 strains did not demonstrate significant antimutagenic properties (less than 30%) against AFB_1_-induced mutagenesis (Figure 4E). Interestingly, ASBP-AH30 and ASBP-AH3 exhibited moderate inhibition (32.49% and 38.74%, respectively) against MeIQ-induced mutagenesis in TA100 under +S9 conditions (Figure 4F). These results suggest that ASBP-AH3 demonstrated the most effective antimutagenic activity, particularly against AF-2, MeIQ, and NaN_3_.

### 3.5. Anticancer Activity of ASBP-AH Fractions in Colon Cancer Cell Lines

**Figure 5:**
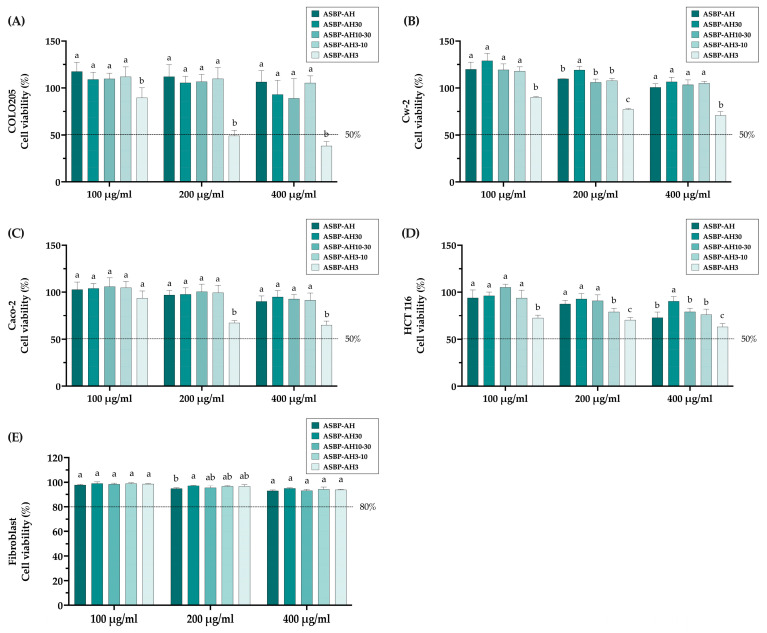
Cytotoxicity of ASBP-AH’s fractions: (**A**–**D**) %Cell viabilities of ASBP-AH’s fractions on various colon cancer cell lines. (**E**) %Cell viabilities of fibroblast cell lines. The values are expressed as mean ± SD (n = 3). According to a one-way ANOVA with Duncan’s multiple range test, a distinct letter indicates a statistically significant difference between groups (*p* < 0.05).

The anticancer activity of ASBP-AH and its fractions against colon cancer cell lines was evaluated using the CCK-8 assay. As shown in Figure 5A–D, the treatment of COLO205, CW-2, Caco-2, and HCT 116 cells with ASBP-AH3 at 400 μg/mL significantly reduced cell viability to 38.19%, 69.53%, 64.97%, and 64.10%, respectively. In contrast, other ASBP-AH fractions at the same concentration showed no effect on cell viability, except HCT116 cells. Specifically, ASBP-AH, ASBP-AH10-30, and ASBP-AH3-10 at 400 µg/mL significantly reduced cell viability to 72.89%, 79.22%, and 76.28%, respectively. Furthermore, ASBP-AH and its fractions had no impact on the viability of human fibroblast cell lines (Figure 5E). These results indicate that ASBP-AH3 is the most effective fraction against colon cancer cells, particularly COLO205 cells, while demonstrating no toxicity to normal cells. Based on these findings, we selected COLO205 cells as the primary model for further investigation into the molecular mechanism.

### 3.6. Microarray Analysis in ASBP-AH3-Treated COLO205 Cells

**Figure 6:**
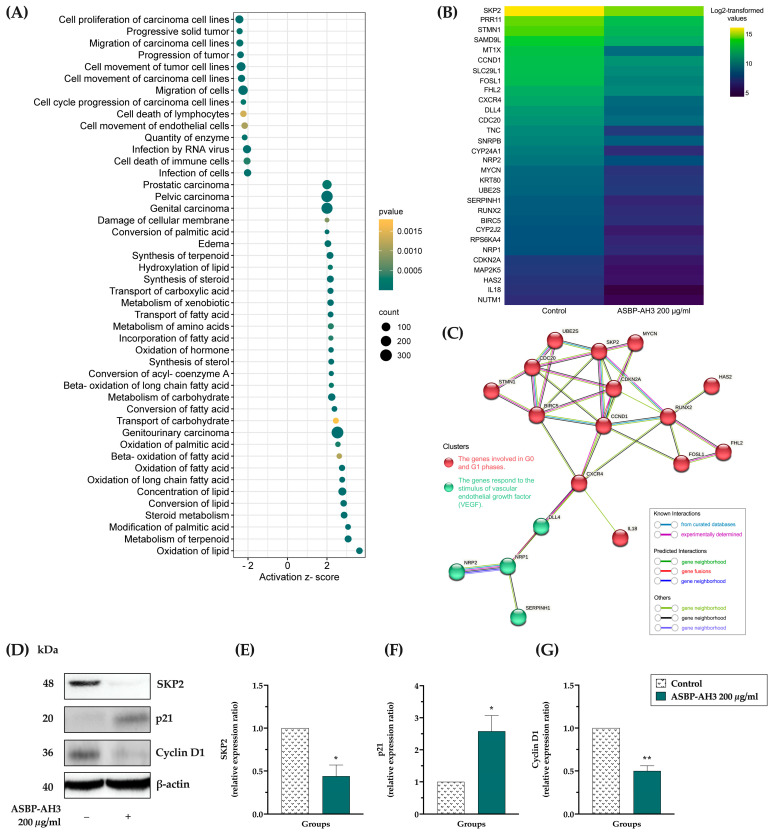
Overview of microarray results and protein expression analysis in ASBP-AH3-treated COLO205 cells: (**A**) the compilation of upregulated (z-score > 2) and downregulated (z-score < −2) pathways identified from the Diseases and Bio Functions dataset following LC_50_ treatment of COLO205 cancer cells with ASBP-AH3; (**B**) the heatmap of log_2_-transformed signal values for inactivated genes from the same dataset; (**C**) the STRING protein–protein interaction network of inactivated genes from the Diseases and Bio Functions dataset; (**D**) Western blot analysis; and (**E**–**G**) expression ratios of SKP2, p21, and cyclin D1 proteins in the ASBP-AH3-treated group compared to control. Data are presented as mean ± SD (n = 3), with differences between groups determined using Student’s *t*-test (* *p* < 0.05, ** *p* < 0.01).

The molecular mechanism underling the anticancer property of ASBP-AH3 on COLO205 cells was investigated using a microarray analysis. Differential gene expressions (DEGs) between ASBP-AH3-treated and untreated cells were analyzed using IPA. A total of 494 genes were significantly differentially expressed (*p* < 0.05 and fold change ≥ 2), with 277 genes upregulated and 217 genes downregulated (Supplementary Data S1). The significant DEGs were subsequently subjected to functional annotation and pathway analysis. As shown in Figure 6A, the most significantly downregulated pathway, based on the Diseases and Bio Functions dataset, was associated with the cell proliferation of carcinoma cell lines (z-score = −2.437). Figure 6B presents a heatmap showing the 30 genes associated with the cell proliferation of carcinoma cell lines that were downregulated. These downregulated genes were further analyzed using the STRING database to construct a protein–protein interaction (PPI) network. Two clusters were identified in the analysis: one comprising 14 genes associated with the G0 and G1 phases of the cell cycle, and another containing 4 genes related to the vascular endothelial growth factor (VEGF) response (Figure 6C). Consequently, the effect of ASBP-AH3 on the expression of cell cycle regulatory proteins was investigated by Western blot analysis in COLO205 cells. As shown in Figure 6D–G, treatment with ASBP-AH3 at 200 μg/mL reduced the expression of cyclin D1 and S-phase kinase-associated protein 2 (SKP2) while upregulating the level of p21. Collectively, these findings suggest that the SKP2/p21/cyclin D1 signaling pathway may play a key role in the ASBP-AH3-mediated regulation of cell viability in COLO205 cells.

### 3.7. Amino Acid Compositions of ASBP-AH Fractions

The functional properties of peptide hydrolysates are strongly influenced by their amino acid composition [40]. Generally, antioxidant peptides with high activity are rich in hydrophobic and aromatic amino acids [23,24,41]. To assess the amino acid profile of ASBP-AH fractions, their composition was analyzed using HPLC, and the results are presented in Table 2. The percentages of essential amino acids (EAAs) and branched-chain amino acids (BCAAs) did not differ significantly among the fractions. Notably, ASBP-AH30 exhibited the highest proportion of aromatic amino acids, which was significantly greater than that in the ASBP-AH group. Furthermore, the size of each fraction was positively correlated with its aromatic amino acid content, which in turn was associated with enhanced antioxidant activity. In contrast, ASBP-AH3 showed the highest percentage of hydrophobic amino acids, with significant differences compared to ASBP-AH. This finding supports the critical role of hydrophobic amino acids in facilitating peptide interaction with and the penetration of cancer cell membranes [42].

### 3.8. Characterization of Peptides Derived from ASBP-AH3

The amino acid sequences of peptides from the ASBP-AH3 fraction, exhibiting promising anticancer activity, were identified using LC-MS/MS. As shown in Table 3, the MWs of these peptides ranged from 707.43 to 1662.96 Da, with hydrophobicity levels varying from 11.1% to 83.3%. Notably, the peptide YFMVLVVMLFHR exhibited the highest hydrophobicity at 83.3%, whereas QQQFDRKNK showed the lowest at 11.1%. Additionally, the distribution of positively charged residues varied considerably; for example, AKAKYK and QQQFDRKNK demonstrated the highest proportions of positively charged residues, at 50.0% and 33.3%, respectively. Notably, the peptides AKAKYK and APLATHGMYK were identified as bioactive anticancer peptides in the BIOPEP database. These results suggest that the ASBP-AH3 fraction contains peptides with a combination of highly hydrophobic residues and significant positive charges, which are likely important for its anticancer activity.

## 4. Discussion

Advancements in biology and biomedicine have led to the discovery and isolation of numerous bioactive peptides from natural animal and plant sources. These peptides are known for their wide range of activities, including antioxidant, anti-inflammatory, antimicrobial, and anticancer properties [1,43]. BSFL are a highly nutritious edible insect, rich in essential nutrients such as fats and proteins [22]. However, the biological properties of bioactive peptide from BSFL protein hydrolysate remain largely unexplored. In this study, we demonstrated that peptide hydrolysates from BSFL exhibit antioxidant, anti-inflammatory, and anticancer activities.

The BSFL used in this study contained a high protein content and relatively low fat content compared to previously reported values [44]. Although fat content has been shown to affect the activity of enzyme hydrolysates [45], the fat in BSFL was removed using hexane extraction prior to the hydrolysis process. Subsequently, the proteins from BSFL were solubilized using an alkaline solution and precipitated with HCl to obtain the ASBP fraction. This method effectively increased the protein content while reducing the fat content in the ASBP. However, more smear bands were observed, although the key MW bands remained detectable. These results are consistent with findings reported by Caligiani et al., which demonstrated that the alkaline extraction of defatted BSFL powder enhances protein recovery and induces protein hydrolysis [37].

Active peptides typically contain between 2 and 20 amino acid residues, and their biological activity is influenced by molecular size, amino acid composition, and sequence, all of which contribute to their structural properties [46]. The functional properties of hydrolysate peptides are determined by several factors, including the protein source, enzyme type, and specific hydrolysis conditions such as pH and temperature [47]. Thus, the optimization of these parameters is essential. Compared to chemical methods or the use of intestinal enzymes, enzymatic hydrolysis using commercial enzymes offers significant advantages, including precise control over the hydrolysis process, enabling the production of peptides with specific and desirable properties [48,49]. In this study, hydrolysis conditions for ASBP were optimized using Alcalase and bromelain, with antioxidant properties evaluated as the endpoint assay. Single Alcalase hydrolysis demonstrated superior antioxidant activity at a concentration of 3% (*w*/*w*) during 4 h of incubation, particularly in the ABTS and DPPH assays, when compared to single bromelain and combination enzyme groups. These results are consistent with findings reported by Xu et al., who demonstrated that Alcalase hydrolysis improved the oxygen radical antioxidant capacity of protein isolates from pigeon pea, lentil, and chickpea compared to bromelain [4]. The DH, an important parameter reflecting hydrolysis efficiency, is closely linked to the MW distribution of peptides [50].

Ultrafiltration effectively fractionates protein hydrolysates to isolate bioactive peptides. Interestingly, ASBP-AH30 (MW >30 kDa) exhibited the strongest antioxidant activity, which decreased with declining fraction size. Aromatic amino acids are generally abundant in the most active antioxidant peptides [23,24,41]. Studies have shown that tyrosine, phenylalanine, and tryptophan contribute to antioxidant activity by donating electrons to free radicals [51]. In this study, ASBP-AH30 displayed the highest percentage of aromatic amino acids compared to the other fractions, further supporting its superior antioxidant potential. In contrast, the ASBP-AH fraction with MWs below 3 kDa (ASBP-AH3) contained the highest percentage of hydrophobic amino acids and demonstrated the strongest anti-inflammatory activity. Consistent with previous studies, it has been found that peptides with hydrophobic amino acids had certain anti-inflammatory regulatory activities [52]. These findings suggest that the MW and amino acid composition of ASBP-AH fractions are key factors in determining their biological activities, including antioxidant and anti-inflammatory properties.

DNA damage is a major driver of cancer development, leading to mutations in oncogenes and tumor suppressor genes, ultimately transforming cells into a malignant state [53]. As a result, identifying compounds that can prevent mutations has become increasingly important. Ames test results showed that ASBP-AH and its fractions exhibited no mutagenic activity, confirming their safety for food and therapeutic applications. Among the tested fractions, ASBP-AH3 demonstrated the strongest antimutagenic activity in non-metabolic (-S9) conditions, with reducing AF-2 mutagenicity in TA98 and NaN_3_ mutagenicity in TA10, suggesting its ability to inhibit direct-acting mutagens. Interestingly, ASBP-AH30 and ASBP-AH3 demonstrated moderate inhibition against MeIQ-induced mutagenesis in TA100 under +S9 conditions. The inclusion of the S9 mix in antimutagenic tests is essential as it mimics the metabolic activation that occurs in living organisms, enabling the evaluation of a compound’s mutagenic potential after metabolic conversion [54]. Notably, the mutagenicity of AF-2 and MeIQ was observed to be greatest in the presence of S9 fractions. The moderate inhibition of MeIQ-induced mutagenesis by ASBP-AH3 and ASBP-AH30 under +S9 conditions suggests that these fractions may reduce the mutagenic effects of MeIQ by modulating its metabolic activation. These findings highlight ASBP-AH3 as the most promising bioactive fraction with potent antimutagenic properties, warranting further investigation into its protective effects against mutagen-induced DNA damage.

Bioactive peptides with low MW have been recognized as potential cancer treatments. In the present study, anticancer activity increased as the MW of ASBP-AH fractions decreased, with ASBP-AH3 showing the strongest effect by effectively inducing colon cancer cell death, particularly in COLO205 cells. This outcome aligns with findings by Jumeri and Kim, who reported that peptides with shorter chain lengths exhibit greater mobility and interaction with cancer cell components, leading to enhanced anticancer activity [55]. Moreover, ASBP-AH3 contains the highest percentage of hydrophobic amino acids, which are key biophysical attributes contributing to its anticancer activity [6]. The combination of hydrophobic and positively charged residues likely enhances anticancer effects by improving membrane interaction, facilitating internalization, and promoting electrostatic interactions with the negatively charged cancer cell surface, aiding cellular uptake [56,57]. Hydrophobic amino acids such as proline and glycine enhance membrane interactions and proteolytic stability [58]. Tryptophan facilitates peptide penetration through the cell membrane, while tyrosine and tryptophan exhibit cytotoxic effects against certain cancer cells [59]. Additionally, phenylalanine increases the affinity of peptides for target cell membranes and enhances cytotoxic activity [60]. Interestingly, the peptides AKAKYK and APLATHGMYK were recognized as bioactive anticancer peptides through the BIOPEP database. Notably, AKAKYK contains a high proportion of hydrophobic amino acids and a significant amount of lysine, which is known to disrupt cell membrane integrity and facilitate membrane penetration, ultimately contributing to its cytotoxic effects on cancer cells [61]. ASBP-AH3 bioactive peptides exhibit a distinct amino acid composition, including a higher proportion of lysine at the C-terminal, which is one of the key characteristics of anticancer peptides [57]. Compared to bioactive peptides derived from other insect sources, such as crickets [41], BSFL demonstrates rapid growth, low environmental impact, and cost-effectiveness [62], making it a promising and sustainable source for bioactive peptide production.

To investigate the molecular mechanisms underlying the action of ASBP-AH3 in COLO205 cells, differential gene expression was analyzed using microarray technology. Functional annotation and pathway analysis revealed that the most significantly downregulated pathway was associated with cell proliferation in carcinoma cell lines. Furthermore, the protein–protein interaction analysis of 30 genes from this pathway was conducted to elucidate the molecular mechanisms by which ASBP-AH3 impacts COLO205 cells. These genes primarily influence the G0 and G1 phases of the cell cycle and the VEGF response, highlighting their role in regulating cancer cell proliferation in COLO205 cells. To confirm these findings, the effect of ASBP-AH3 on the expression of G1 cell cycle regulatory proteins was examined. Treatment with ASBP-AH3 led to a reduction in the expression of SKP2 and cyclin D1 proteins, while the level of the cell cycle inhibitor p21 was significantly increased. SKP2 is a critical regulator of cell cycle progression and is frequently overexpressed in various cancers, including colon cancers [63]. This overexpression contributes to tumor growth by targeting cell cycle inhibitors such as p21 for degradation [64]. Additionally, the loss of SKP2 function can lead to decreased cyclin D1 levels as a result of the reduced ubiquitination and degradation of p21 [65]. These findings support our results, suggesting that ASBP-AH3 inhibits COLO205 cell proliferation by modulating the expression of cell cycle regulatory proteins. Specifically, ASBP-AH3 reduces SKP2 and cyclin D1 levels while upregulating p21, thereby disrupting the cell cycle and suppressing tumor growth. Although ASBP-AH3 shows promising therapeutic potential, several factors must be considered before clinical application, including safety concerns, allergenicity, and public acceptance of insect-based products, which our study addresses by demonstrating no toxicity in normal fibroblast cell lines. However, further research, including *in vivo* studies using appropriate animal models like xenograft or genetically modified mice, is needed to assess the long-term safety, bioavailability, efficacy, and antioxidant effects of these peptides in a more physiologically relevant context, ultimately facilitating the safe and widespread use of BSFL-derived peptides in therapeutic applications.

## 5. Conclusions

In this study, ASBP-AH, hydrolyzed at 3% (*w*/*w*) for 4 h, exhibited the highest antioxidant activity. ASBP-AH was fractionated by MW, with ASBP-AH30 (MW > 30 kDa) showing the strongest antioxidant activity, while ASBP-AH3 (MW < 3 kDa) demonstrated superior antimutagenic, anti-inflammatory, and anticancer effects, particularly against COLO205 cells. Mechanistic analysis revealed that ASBP-AH3 induces cytotoxicity by modulating the SKP2/p21/cyclinD1 pathway, with peptides’ hydrophobic and charged amino acids facilitating their interaction with cellular membranes. These findings highlight the potential of BSFL-derived peptides for cancer chemoprevention; however, further *in vivo* studies are essential to explore their clinical applicability, as well as their bioavailability and potential metabolism.

## Figures and Tables

**Table 1:** Proximate analysis of BSFL, BSFL-H and ASBP.

Samples	Proximate Compositions of BSFL (%)			
	Crude Protein	Fat	Ash	Moisture
BSFL	55.45 ± 0.02 ^c^	18.45 ± 0.10 ^a^	7.77 ± 0.63 ^b^	5.62 ± 0.03 ^c^
BSFL-H	64.53 ± 0.31 ^b^	6.66 ± 0.52 ^b^	8.90 ± 0.44 ^a^	10.31 ± 0.08 ^a^
ASBP	77.26 ± 0.03 ^a^	2.21 ± 0.20 ^c^	9.35 ± 0.05 ^a^	8.48 ± 0.16 ^c^

Values are expressed as mean ± SD (n = 3). Statistical significance was determined using one-way ANOVA followed by Duncan’s multiple range test. Different superscript letters indicate significant differences between groups (*p* < 0.05). Abbreviations: BSFL-H, BSFL defatted by hexane; and ASBP, alkali-soluble BSFL protein.

**Table 2:** The percent of amino acid compositions consisted of ASBP-AH and its fraction detected by RP-HPLC.

Amino Acid Groups	Amino Acid Compositions (% Amino Acid/g Sample)				
	ASBP-AH	ASBP-AH30	ASBP-AH10-30	ASBP-AH3-10	ASBP-AH3
Positively Charged Side-Chain Amino Acid					
Arginine (Arg)	1.92 ± 0.04 ^a^	1.96 ± 0.47 ^a^	1.92 ± 0.39 ^a^	1.87 ± 0.33 ^a^	1.54 ± 0.12 ^a^
Histidine (His)	2.02 ± 0.23 ^a^	2.15 ± 0.10 ^a^	2.13 ± 0.12 ^a^	2.13 ± 0.20 ^a^	2.22 ± 0.01 ^a^
Lysine (Lys)	6.05 ± 0.91 ^a^	5.70 ± 0.16 ^a^	6.22 ± 0.50 ^a^	5.52 ± 0.89 ^a^	5.59 ± 0.24 ^a^
Negatively Charged Side-Chain Amino Acid					
Asparagine (Asp)	13.00 ± 5.26 ^a^	12.12 ± 1.66 ^a^	12.08 ± 1.95 ^a^	12.30 ± 2.88 ^a^	8.98 ± 1.63 ^a^
Glutamic acid (Glu)	14.61 ± 1.01 ^a^	10.53 ± 0.46 ^a^	10.64 ± 0.50 ^a^	12.84 ± 4.68 ^a^	11.08 ± 1.27 ^a^
Polar Uncharged Side-Chain Amino Acid					
Serine (Ser)	6.32 ± 0.67 ^a^	5.77 ± 0.88 ^a^	5.63 ± 0.45 ^a^	6.27 ± 0.24 ^a^	5.22 ± 0.39 ^a^
Threonine (Thr)	3.56 ± 0.36 ^a^	3.25 ± 0.41 ^a^	3.39 ± 0.28 ^a^	3.27 ± 0.60 ^a^	3.61 ± 0.32 ^a^
Hydroxyproline (Hyp)	0.29 ± 0.09 ^a^	0.14 ± 0.14 ^ab^	0.17 ± 0.09 ^ab^	0.10 ± 0.06 ^b^	0.10 ± 0.07 ^b^
Hydrophobic Amino Acid					
Alanine (Ala)	9.37 ± 0.98 ^b^	12.32 ± 1.50 ^ab^	12.11 ± 1.68 ^ab^	11.60 ± 2.72 ^ab^	15.59 ± 2.79 ^a^
Methionine (Met)	2.66 ± 0.42 ^a^	2.19 ± 0.87 ^a^	2.38 ± 0.69 ^a^	1.98 ± 0.24 ^a^	2.23 ± 0.54 ^a^
Glycine (Gly)	8.37 ± 1.81 ^a^	8.70 ± 1.40 ^a^	8.79 ± 1.27 ^a^	8.70 ± 0.80 ^a^	9.47 ± 0.52 ^a^
Proline (Pro)	6.19 ± 0.83 ^a^	6.29 ± 1.07 ^a^	7.24 ± 1.51 ^a^	7.48 ± 1.19 ^a^	6.81 ± 0.17 ^a^
BCAA					
Valine (Val)	6.02 ± 0.31 ^a^	6.68 ± 0.20 ^a^	6.42 ± 0.04 ^a^	6.15 ± 0.37 ^a^	6.36 ± 0.67 ^a^
Isoleucine (Ile)	4.24 ± 0.19 ^a^	3.98 ± 0.56 ^a^	3.79 ± 0.60 ^a^	3.50 ± 0.03 ^a^	3.65 ± 0.42 ^a^
Leucine (Leu)	7.42 ± 0.59 ^a^	7.27 ± 1.16 ^a^	6.79 ± 1.01 ^a^	6.43 ± 0.39 ^a^	7.72 ± 0.57 ^a^
Aromatic AA					
Phenylalanine (Phe)	3.12 ± 0.35 ^a^	3.78 ± 0.56 ^a^	3.59 ± 0.42 ^a^	3.30 ± 0.25 ^a^	3.16 ± 0.17 ^a^
Tyrosine (Tyr)	4.86 ± 1.46 ^b^	7.18 ± 1.72 ^a^	6.71 ± 0.98 ^ab^	6.57 ± 0.35 ^ab^	6.67 ± 0.46 ^ab^
EAA	36.99 ± 1.42 ^a^	36.95 ± 1.79 ^a^	36.63 ± 1.39 ^a^	34.14 ± 2.81 ^a^	36.08 ± 1.37 ^a^
Hydrophobic AA	52.23 ± 4.67 ^b^	58.38 ± 0.68 ^ab^	57.82 ± 0.81 ^ab^	55.70 ± 5.85 ^ab^	61.67 ± 1.91 ^a^
Aromatic AA	7.98 ± 1.11 ^b^	10.95 ± 2.28 ^a^	10.30 ± 1.39 ^ab^	9.87 ± 0.60 ^ab^	9.84 ± 0.64 ^ab^
BCAA	17.67 ± 0.48 ^a^	17.93 ± 1.51 ^a^	17.00 ± 1.57 ^a^	16.07 ± 0.78 ^a^	17.74 ± 1.67 ^a^

Abbreviations: AA, amino acid; BCAA, branched-chain amino acid; and EAA, essential amino acid. EAAs include His, Arg, Thr, Val, Met, Ile, Leu, Phe, and Lys. Hydrophobic AAs include Ala, Met, Gly, Pro, Val, Ile, Leu, Phe, and Tyr. Aromatic AAs include Phe and Tyr; BCAAs include Ile, Leu, and Val. Positively charged side-chain AAs include Arg, His, and Lys. Data are presented as mean ± SD (n = 3). One-way ANOVA followed by Duncan’s multiple range test is applied, with different letters indicating significant differences between groups (*p* < 0.05).

**Table 3:** Bioactive peptides derived from ASBP-AH3 are characterized using LC-MS/MS.

Peptides	Amino Acid Sequence	Mass (Da)	%Hydrophobic AA	%Positive Charge Side Chains AA
AKAKYK	Ala-Lys-Ala-Lys-Tyr-Lys	707.4330	50%	50%
GWWTKK	Gly-Trp-Trp-Thr-Lys-Lys	804.4282	33%	33%
TLVPVMDLK	Thr-Leu-Val-Phe-Val-Met-Asp-Leu-Lys	1014.5832	67%	11%
KNVSLVMPK	Lys-Asn-Val-Ser-Leu-Val-Met-Phe-Lys	1014.5945	56%	22%
QQQFDRKNK	Gln-Gln-Gln-Phe-Asp-Arg-Lys-Asn-Lys	1190.6155	11%	33%
YFMVLVVMLFHR	Tyr-Phe-Met-Val-Leu-Val-Val-Met-Leu-Phe-His-Arg	1553.8301	83%	17%
NEVKFVYR	Asn-Glu-Val-Lys-Phe-Val-Tyr-Arg	1053.5608	50%	25%
APLATHGMYK	Ala-Pro-Leu-Ala-Thr-His-Gly-Met-Tyr-Lys	1087.5535	50%	20%
FALSLLMMR	Phe-Ala-Leu-Ser-Lys-Lys-Met-Met-Arg	1080.5823	56%	33%
TGPVEDCAK	Thr-Gly-Pro-Val-Glu-Asp-Cys-Ala-Lys	918.4131	22%	11%
FYLPVTMWCDK	Phe-Tyr-Leu-Pro-Val-Thr-Met-Trp-Cys-Asp-Lys	1401.6526	55%	9%
VDPLLSNVALSAPLVR	Val-Asp-Pro-Lys-Lys-Ser-Asn-Val-Ala-Lys-Ser-Ala-Pro-Lys-Val-Arg	1662.9668	31%	31%
KDVGLTYFDFK	Lys-Asn-Val-Gly-Leu-Thr-Tyr-Phe-Asp-Phe-Lys	1331.6760	45%	18%
HALLTSER	His-Ala-Lys-Lys-Thr-Ser-Glu-Arg	925.4981	13%	50%
APLAYSTPLLK	Ala-Pro-Leu-Ala-Tyr-Ser-Thr-Pro-Leu-Leu-Lys	1172.6804	55%	9%
QSVNHK	Gln-Ser-Val-Asn-His-Lys	711.3664	17%	33%

## Data Availability

The original contributions presented in this study are included in the article and Supplementary Material. Further inquiries can be directed to the corresponding author.

## Supplementary Materials

The following supporting information can be downloaded at: https://www.mdpi.com/article/10.3390/nu17040645/s1, Date S1: List of significantly differentially expressed genes, Data S2: Upregulated and downregulated pathway analysis.
